# PKM2–c-Myc–Survivin Cascade Regulates the Cell Proliferation, Migration, and Tamoxifen Resistance in Breast Cancer

**DOI:** 10.3389/fphar.2020.550469

**Published:** 2020-09-08

**Authors:** Pian Yu, Ao-xue Li, Xi-sha Chen, Min Tian, Hai-yan Wang, Xin-luan Wang, Yi Zhang, Kuan-song Wang, Yan Cheng

**Affiliations:** ^1^ Department of Pharmacy, The Second Xiangya Hospital, Central South University, Changsha, China; ^2^ Xiangya School of Pharmaceutical Sciences, Central South University, Changsha, China; ^3^ Translational Medicine R&D Center, Institute of Biomedical and Health Engineering, Shenzhen Institutes of Advanced Technology, Chinese Academy of Sciences, Shenzhen, China; ^4^ Department of Pharmacology, College of Pharmaceutical Sciences, Soochow University, Suzhou, China; ^5^ Department of Pathology, Xiangya Hospital, Central South University, Changsha, China

**Keywords:** breast cancer cells, M2 isoform of pyruvate kinase, tamoxifen resistance, c-Myc, survivin

## Abstract

The M2 isoform of pyruvate kinase (PKM2), as a key glycolytic enzyme, plays important roles in tumorigenesis and chemotherapeutic drug resistance. However, the intricate mechanism of PKM2 as a protein kinase regulating breast cancer progression and tamoxifen resistance needs to be further clarified. Here, we reported that PKM2 controls the expression of survivin by phosphorylating c-Myc at Ser-62. Functionally, PKM2 knockdown suppressed breast cancer cell proliferation and migration, which could be rescued by overexpression of survivin. Interestingly, we found that the level of PKM2 expression was upregulated in the tamoxifen resistant breast cancer cells MCF-7/TAMR, and knockdown of PKM2 sensitized the cells to 4-hydroxytamoxifen (4OH-T). In addition, the elevated level of PKM2 correlates with poor relapse-free survival in breast cancer patients treated with tamoxifen. Overall, our findings demonstrated that PKM2–c-Myc–survivin cascade regulated the proliferation, migration and tamoxifen resistance of breast cancer cells, suggesting that PKM2 represents a novel prognostic marker and an attractive target for breast cancer therapeutics, and that PKM2 inhibitor combined with tamoxifen may be a promising strategy to reverse tamoxifen resistance in breast cancer patients.

## Introduction

Pyruvate kinase isoform M2 (PKM2), one of the isoenzymes of pyruvate kinase (PK) (Yang and Lu, 2013), is a key glycolytic enzyme overexpressed in cancer cells (Luo and Semenza, 2012), which controls the terminal rate-limiting step of glycolysis by catalyzing the transform of a phosphate group from phosphoenolpyruvate (PEP) to adenosine diphosphate (ADP) (Hamanaka and Chandel, 2011). Previous reports suggested that PKM2 affects cell proliferation, migration, invasion, apoptosis, and cell cycle progression of tumors, including breast cancer, prostate cancer, myeloma, liver cancer, lung cancer, and pancreatic cancer (Stetak et al., 2007; Christofk et al., 2008; Yang et al., 2012; Jiang et al., 2014; Christensen et al., 2015; Azoitei et al., 2016; Matsuda et al., 2016; Shen et al., 2016; Guo et al., 2017). Over the years, there have been increasing evidence pointing to the non-glycolytic function of PKM2 in tumor cells. For instance, PKM2 binds to and transactivates Y333-phosphorylated *β*-catenin to promote tumor cell proliferation (Yang et al., 2011); PKM2 promotes tumorigenesis by directly phosphorylating histone H3 at threonine 11 under EGFR activation (Yang et al., 2012); A recent study has demonstrated that nuclear PKM2 activates transcription of MEK5 by phosphorylating STAT3 at tyrosine 705 in colon cancer cells (Gao et al., 2012). However, the role of PKM2 as a protein kinase in the regulation of tumor progression in breast cancer remains to be further identified. In addition, studies have shown that PKM2 is highly correlated with drug resistance. For example, down-regulation of PKM2 by shikonin, an inhibitor of PKM2, re-sensitized the drug resistant bladder cancer cells to cisplatin (Wang et al., 2018); PKM2 expressions were positively associated with gefitinib resistance in colorectal cancer cells, and PKM2 knockdown increased gefitinib efficacy (Li et al., 2015). It has been demonstrated recently that NAMPT promotes tamoxifen resistance *via* regulation of the PKM2 translocation (Ge et al., 2019). However, the specific mechanism and role of PKM2 in regulating breast cancer tamoxifen resistance remains unknown.

c-Myc is one of the most activated oncogenes and is associated with the initiation and progression of human cancer (Dang, 1999; Nesbit et al., 1999). c-Myc was defined as an oncoprotein associated with DNA replication, transcription or RNA splicing. It has been demonstrated that c-Myc can regulate the transcription of survivin (encoded by the gene *BIRC5*), an essential member of the inhibitor of apoptosis protein (IAP) family, playing an important role in tumorigenesis (Cosgrave et al., 2006; Fang et al., 2009; Papanikolaou et al., 2011; Chen et al., 2016; Garg et al., 2016; Haque et al., 2017). Studies have shown that survivin is dramatically overexpressed in various of tumors, such as breast cancer, colon cancer, and lung cancer, and promotes the proliferation and metastasis of tumor cells (Kawasaki et al., 1998; Monzo et al., 1999; Suzuki et al., 2000; Tanaka et al., 2000; Marioni et al., 2006; McKenzie et al., 2010; Kedinger et al., 2013; Ma et al., 2016; Yang et al., 2016; Liu et al., 2019). Furthermore, the elevated survivin expression in cancer patients reveals a poor prognosis and high mortality rate (Ma et al., 2016). Survivin is usually expressed in tumor tissue, but infrequently measured in normal differentiated adult tissues. Therefore, survivin is a prospective target for the diagnosis and therapy of cancer.

In this study, we investigated the action of PKM2 as a protein kinase in the regulation of proliferation and migration of breast cancer cells. We demonstrated for the first time that PKM2 regulated the expression of survivin by interacting with c-Myc and phosphorylating c-Myc at Ser-62. In addition, we found that PKM2 was upregulated in tamoxifen resistant breast cancer cells, and PKM2 downregulation enhanced cell sensitivity to tamoxifen in both of MCF-7 and MCF-7/TAMR cells. Therefore, targeting PKM2–c-Myc–survivin pathway may provide a new strategy for inhibiting breast cancer cell proliferation and migration and for reversing tamoxifen resistance.

## Materials and Methods

### Cell Lines and Culture

The human breast cancer cell lines MCF-7 and MDA-MB-231 were cultured in Dulbecco’s modified Eagle’s medium (DMEM)/High glucose medium supplemented with 10% fetal bovine serum (Gibco). The tamoxifen resistant cell line MCF-7/TAMR was purchased from China Medical University. The MCF-7/TAMR cells were cultured in DMEM supplemented with 10% fetal bovine serum (FBS) and 0.4 μM 4OH-Tamoxifen. The cells were maintained at 37°C in a humidified atmosphere with 5% CO_2_.

### Reagents and Antibodies

4-hydroxytamoxifen (4OH-T) was purchased from Sigma. MG132 and CHX were purchased from Selleck. Rabbit monoclonal antibodies against PKM2, c-Myc, and survivin were purchased from Cell Signaling Technologies. Anti-phospho-c-Myc (Ser62) was purchased from Abcam. Anti-*β*-actin, GST, and Flag antibodies were purchased from Proteintech.

### Transfection

The siRNA duplexes targeting PKM2 and c-Myc were purchased from Ribobio. Non-targeting siRNA was used as a control. Transfection of siRNA was accomplished according to the manufacturer’s protocol. In brief, cells in the exponential phase of growth were plated in 6-well plates at 1 × 10^5^ cells per well, grown for 24 h, then transfected with siRNA using Lipofectamine 2000 (Invitrogen) and OPTI-MEM reduced serum medium. For plasmid transfection, cells were transfected with a mixture of GST–PKM2 or His-c-Myc plasmid and Lipofectamine 2000 (Invitrogen) in OPTI-MEM reduced serum medium.

### Quantitative Real-Time Polymerase Chain Reaction (qRT-PCR)

Total RNA was isolated from MCF-7 and MDA-MB-231 cells using Trizol reagent (Biotech) according to the manufacturer’s instructions. The quality, quantity, and integrity of RNA were measured by Nanodrop2000 spectrophotometer. The TaqMan high-capacity cDNA Kit (Takara) was used for the reverse transcription of mRNA. GAPDH acted as an internal control relative to survivin. Primers were designed and synthesized by Sangon Biotech.

### Western Blot and Immunoprecipitation

Cells were washed with PBS and lysed by RIPA buffer (Beyotime, Haimen, China) supplemented with a phosphatase inhibitor cocktail and a protease inhibitor cocktail (Selleck). The concentrations of protein were quantified with a BCA protein assay kit (Beyotime, Haimen, China). The proteins were isolated by 10% SDS-PAGE and transferred to a PVDF membrane. The PVDF membranes were incubated with primary antibody in 5% skim milk with Tween 20 for 1 h at room temperature. Membranes were then incubated with anti-rabbit or anti-mouse secondary antibody at room temperature for 1 h. Detection was completed by chemiluminescence using an ECL reagent. For immunoprecipitation under denaturing conditions, proteins were extracted using regular immunoprecipitation. The beads were washed, and then resolved by 10% SDS-PAGE. The proteins were visualized by Western Blot.

### Protein Half-Life Assay

To examine c-Myc protein stability, siRNA targeting PKM2 was transfected into MCF-7 and MDA-MB-231cells. The cells were treated with cycloheximide (CHX; 20 μg/ml; Amresco) and were harvested at the indicated time points for immunoblotting.

### Cell Viability Assay

Cell viability was measured by the Cell Counting Kit-8 (CCK-8) assay according to the manufacturer’s protocol. Briefly, cells were cultured in 96-well plates at 5 × 10^3^ cells per well. After treatment, cells were incubated with 10 μl CCK-8 reagent for 2 h at 37°C with 5% CO_2_. The results were measured at 450 nm wave length.

### EdU Assay

Proliferating cells were stained using the Cell Light EdU DNA Cell Proliferation Kit (Ribobio). Cells were exposed with 50 μmol/L of EdU for 2 h at 37°C. After fixing with 4% paraformaldehyde for 15 min, cells were treated with 0.5% Triton X-100 for 30 min and washed with PBS three times. Then, cells were exposed to 100 μl 1× Apollo^®^ reaction cocktail for 30 min and incubated with 5 μg/ml of Hoechst 33342 to stain the cell nucleus for 30 min. Images were visualized under a fluorescent microscope.

### Wound Healing Assay

After siRNA and plasmid were transfected into MCF-7 and MDA-MB-231 cells, the cells were seeded in 6-well plates at 2 × 10^5^ cells per well and grown overnight. A wound was then made in the cell culture by scratching on the cell layer with a sharp tip, then incubation for a further 48 h in serum-free medium. The gap created by the wound was then detected under a microscope to offer an indication of the wound-healing capability of the cells.

### Migration and Invasion Assays

Migration and invasion were evaluated using 24-well chemotaxis chambers (Costar, #3422, 8 μm pore size). The cells were washed twice with phosphate-buffered saline, resuspended in 100 μl serum-free medium and added into the upper chambers. The lower chambers were ﬁlled with 600 μl medium containing 10% fetal bovine serum. For the migration assay, after incubation for 24 h, the cells that had migrated through the membrane were stained and counted. For the invasion assay, the cells were incubated for 48 h in the upper chamber coated with a mixture of serum-free medium and Matrigel (3:1; BD Biosciences).

### Cell Apoptosis Assays

MCF-7 cells transfected with siRNA were cultured for 72 h and harvested by centrifugation. Cell apoptosis assay was performed by Annexin V-FITC/PI Staining Kit (Mbchem) according to the manufacturer’s instructions.

### Soft Agar Colony Formation Assay

MCF-7/TAMR cells transfected with si-PKM2 or si-NT were seeded on top of 1.2% agar in the RPMI-1640 medium containing 10% FBS with 0.7% agar (Bioweste) in 24-well plate. After 10 days, the clones were observed with a microscope and photographed. Three independent experiments were quantified using Image J. The silencing effects were detected by Western Blot.

### Kaplan–Meier Survival Analysis

The relapse-free survival of patients with tamoxifen-exposed ER+ breast cancer stratiﬁed by PKM2 expression levels (low and high) were evaluated using Kaplan–Meier analysis from a large publicly available clinical breast cancer microarray online database and web tool (http://kmplot.com/analysis/).

### Data Analysis

Data were analyzed by unpaired two-tailed Student’s t-test. All experiments were performed at least three times. Differences between groups were considered statistically significant at *p <*0.05. Graphpad Prism software (version 6.0) was used for analysis.

## Results

### PKM2 Promotes Cell Proliferation and Migration, and Its High Expression Is Correlated With Poor Prognosis in Human Breast Cancer

**Figure 1:**
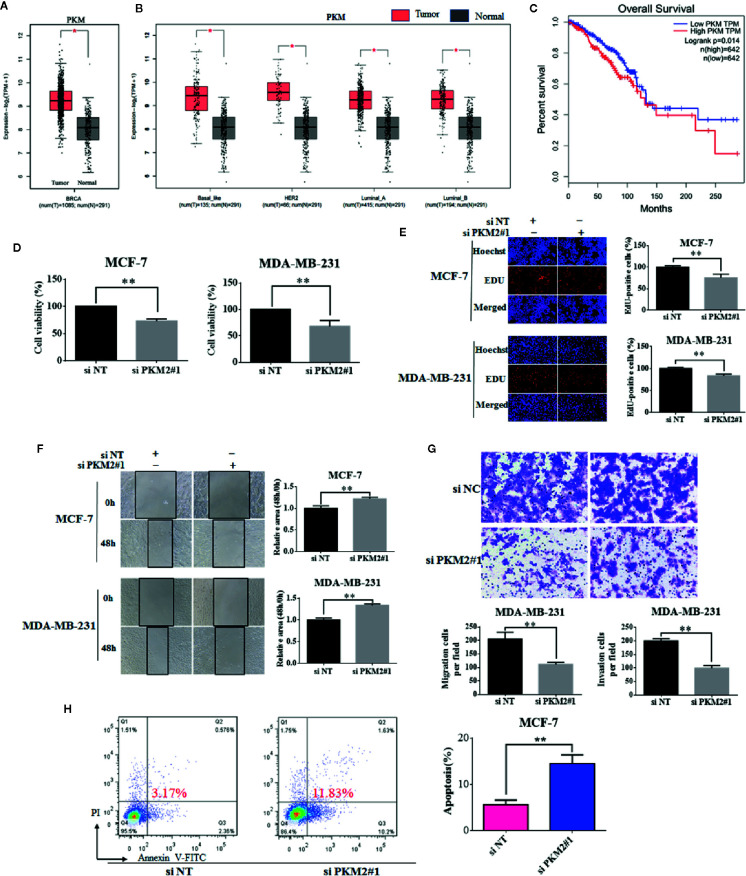
PKM2 promotes cell proliferation and migration, and its high expression is correlated with poor prognosis in human breast cancer. **(A)** GEPIA analysis showed the expression levels of PKM2 between breast cancer and normal tissues. **(B)** GEPIA analysis showed the expression levels of PKM2 in breast cancer subtype tissues compared to normal tissues. **(C)** Survival analysis with auto select best cutoff values of PKM2 expression for breast cancer from TCGA datasets. The MCF-7 or MDA-MB-231 cells were transfected with siPKM2 or non-targeting siRNA. **(D)** Cell viability was measured by CCK-8 reagent. **(E)** Cell proliferation ability was measured by EdU. Magnification, ×200. **(F)** Migration ability was detected by wound healing assay, the black line indicates the edge of migrating cells at a given time point. Data were shown as the mean ± SD of three independent experiments (*p < 0.05, **p < 0.01). **(G)** MDA-MB-231 cells were transfected with non-targeting siRNA or PKM2 siRNA. The migration (left) and invasion (right) ability of cells were measured by transwell assays. Migration cells were incubated for 24 h, invasion cells were incubated for 48 h in the upper chambers coated with Matrigel (*p < 0.05, **p < 0.01). **(H)** MCF-7 cells were transfected with non-targeting siRNA or PKM2 siRNA. Indicated cells were stained with Annexin V/PI, and the percentage of apoptotic cells was assessed by flow cytometry (*p < 0.05, **p < 0.01).

Firstly, we compared the expressions of PKM2 between normal and breast cancer tissues using a large publicly available online database-GEPIA (http://gepia.cancer-pku.cn/). As shown in **Figure 1A**, PKM2 was significantly up-regulated in breast cancer tissues compared to normal tissues. We further analyzed the expressions of PKM2 in different types of breast cancer and found that PKM2 was significantly up-regulated in Basal-like, ER+, HER2+ breast cancer tissues compared to normal tissues (**Figure 1B**). Then, we further analyzed the expressions of PKM2 in ER+ and triple negative breast cancer by TCGA and found that there was no significant difference in the levels of PKM2 between ER+ and triple negative breast cancer tissues (**Supplemental Figure 1A**). We also measured the protein expressions of PKM2 in breast cancer cell lines. Consistently, PKM2 has similar expression in ER+ and ER− breast cancer cells (**Supplemental Figure 1B**). Next, we analyzed the survival rate using GEPIA. The overall survival was lower in patients with high PKM2 expression, suggesting that PKM2 overexpression indicates a high risk of recurrence in breast cancer patients (**Figure 1C**). To further investigate the potential oncogenic role of PKM2 in breast cancer, we knocked down PKM2 and measured the cell viability and proliferation ability in MCF-7 and MDA-MB-231 cells. **Figures 1D, E** show that PKM2 knockdown markedly reduced the viability and proliferation of breast cancer cells. To explore the roles of PKM2 in breast cancer metastasis, we detected the effect of PKM2 on cell migration by wound healing assay. As shown in **Figure 1F**, knockdown of PKM2 markedly reduced the migration of breast cancer cell lines MCF-7 and MDA-MB-231 (**Figure 1F**). In addition, PKM2 knockdown also significantly reduced the migration and invasion of MDA-MB-231 cells using transwell assays (**Figure 1G**). We further examined the potential role of PKM2 in regulating cellular apoptosis. There was increased cellular apoptosis in PKM2 knockdown cells as compared with siRNA control cells (**Figure 1H**). These data suggest that PKM2 is necessary for cell proliferation and migration, and PKM2 may be an important prognostic factor in breast cancer patients.

### PKM2 Promotes Breast Cancer Progression Through Increasing Survivin mRNA and Protein Expressions

**Figure 2:**
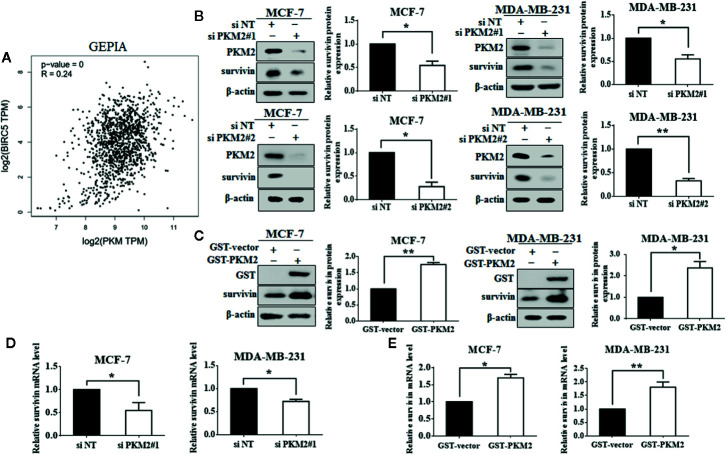
PKM2 regulates survivin expression. **(A)** The correlation between PKM2 and survivin predicted by GEPIA. **(B)** MCF-7 or MDA-MB-231 cells were transfected with either the non-targeting siRNA or PKM2 siRNA. PKM2 and survivin protein levels were measured by immunoblotting. *β*-actin served as a loading control. **(C)** MCF-7 or MDA-MB-231 cells were transfected with either the empty vector plasmid or GST-PKM2 plasmid. GST and survivin protein levels were measured by immunoblotting. *β*-actin served as a loading control. **(D)** MCF-7 or MDA-MB-231 cells were transfected with either the non-targeting siRNA or PKM2 siRNA. PKM2 and survivin mRNA levels were detected by qRT-PCR. GAPDH served as a loading control. **(E)** MCF-7 or MDA-MB-231 cells transfected with either the empty vector plasmid or GST-PKM2 plasmid, PKM2 and survivin mRNA levels were detected by qRT-PCR. GAPDH served as a loading control. Data were shown as the mean ± SD of three independent experiments (*p < 0.05, **p < 0.01).

**Figure 3:**
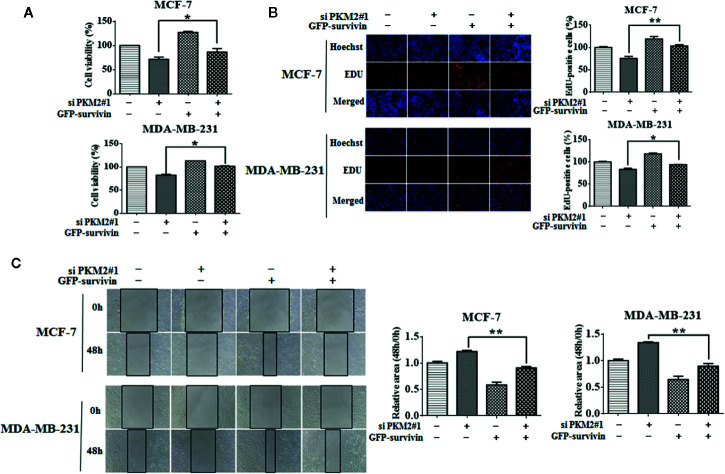
PKM2 promotes breast cancer progression through survivin. MCF-7 or MDA-MB-231 cells were transfected with either the non-targeting siRNA or PKM2 siRNA, followed by transfection with either the empty vector plasmid or GFP-survivin plasmid. **(A)** Cell viability was measured by CCK-8 reagent. **(B)** Cell proliferation ability was measured by EdU. Magnification, ×200. **(C)** Migration ability was detected by wound healing assay, the black line indicates the edge of migrating cells at a given time point. Data were shown as the mean ± SD of three independent experiments (*p < 0.05, **p < 0.01).

Next, we are interested in the molecular mechanism by which PKM2 promotes cell proliferation. PKM2 was predicted to be positively associated with survivin in mRNA level using a publicly online database-GEPIA (**Figure 2A**). Survivin is well known as a member of the inhibitor of apoptosis protein family, which is crucial for the proliferation and migration of breast cancer cells (Tanaka et al., 2000). We found that knockdown of PKM2 led to a significant decrease in survivin expressions at both protein and mRNA (**Figures 2B, D**). Conversely, ectopic overexpression of PKM2 remarkably increased survivin expressions at both protein and mRNA (**Figures 2C, E**). Collectively, our results suggest that PKM2 promotes the expression of survivin by regulating transcription. Next, we further investigated whether survivin mediated the regulatory effect of PKM2 on breast cancer progression. MCF-7 or MDA-MB-231 cells were transfected with PKM2 siRNA, followed by transfection with GFP-survivin plasmid, then the cell viability, proliferation ability, and migration capacity were tested. We found that the inhibition of cell viability, proliferation and migration by PKM2 knockdown was rescued by survivin overexpression in MCF-7 and MDA-MB-231 cells (**Figures 3A–C**). Together, these experiments suggest that PKM2 promotes breast cancer cell proliferation and migration through increasing survivin transcription.

### PKM2 Activates Transcription of Survivin Through c-Myc

**Figure 4:**
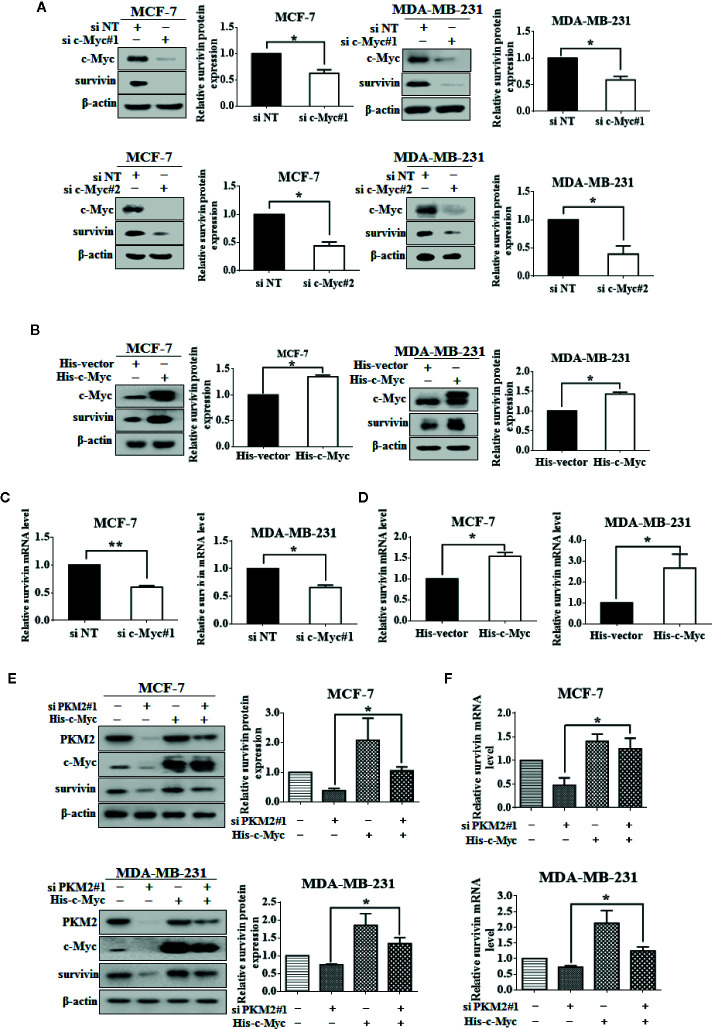
c-Myc regulates PKM2-mediated survivin expression. **(A)** MCF-7 or MDA-MB-231 cells were transfected with either the non-targeting siRNA or c-Myc siRNA. c-Myc and survivin protein levels were measured by immunoblotting. *β*-actin served as a loading control. **(B)** MCF-7 or MDA-MB-231 cells were transfected with either the empty vector plasmid or His-c-Myc plasmid. c-Myc and survivin protein levels were measured by immunoblotting. *β*-actin served as a loading control. **(C)** MCF-7 or MDA-MB-231 cells were transfected with either the non-targeting siRNA or c-Myc siRNA. Survivin mRNA level was detected by qRT-PCR. GAPDH served as a loading control. **(D)** MCF-7 or MDA-MB-231 cells were transfected with either the empty vector plasmid or His-c-Myc plasmid. Survivin mRNA level was detected by qRT-PCR. GAPDH served as a loading control. **(E, F)** MCF-7 or MDA-MB-231 cells were respectively transfected with either the non-targeting siRNA or PKM2 siRNA, and then transfected with either the empty vector plasmid or His-c-Myc plasmid. **(E)** Survivin protein level was detected by immunoblotting. *β*-actin served as a loading control. **(F)** Survivin mRNA level was detected by qRT-PCR. GAPDH served as a loading control. Data were shown as the mean ± SD of three independent experiments (*p < 0.05, **p < 0.01).

We next sought to understand how PKM2 regulates survivin expression. Sequence analyses did not show any known DNA binding domain/motifs in PKM2 (Gao et al., 2012). One of the possibilities is that PKM2 may regulate the activation of a particular transcription factor (Harris et al., 2012). c-Myc is a known transcription factor of survivin (Cosgrave et al., 2006; Fang et al., 2009). Consistent with the previous reports, we verified the regulatory effect of c-Myc on survivin. As shown in **Figures 4A, C**, c-Myc knockdown led to reduced protein and mRNA levels of survivin in MCF-7 and MDA-MB-231 cells. Overexpression of c-Myc increased survivin expression at both mRNA and protein levels (**Figures 4B, D**). We next tested whether PKM2 activated the transcription of survivin through c-Myc. MCF-7 and MDA-MB-231 cells were transfected with PKM2 siRNA, and then transfected with His-c-Myc plasmid. The decrease of survivin at protein and mRNA levels caused by knockdown of PKM2 could be rescued by exogenous c-Myc (**Figures 4E, F**). These results show that PKM2 promotes the transcription of survivin through c-Myc.

### PKM2 Interacts With c-Myc and Stabilizes c-Myc by Phosphorylating It at Ser62

**Figure 5:**
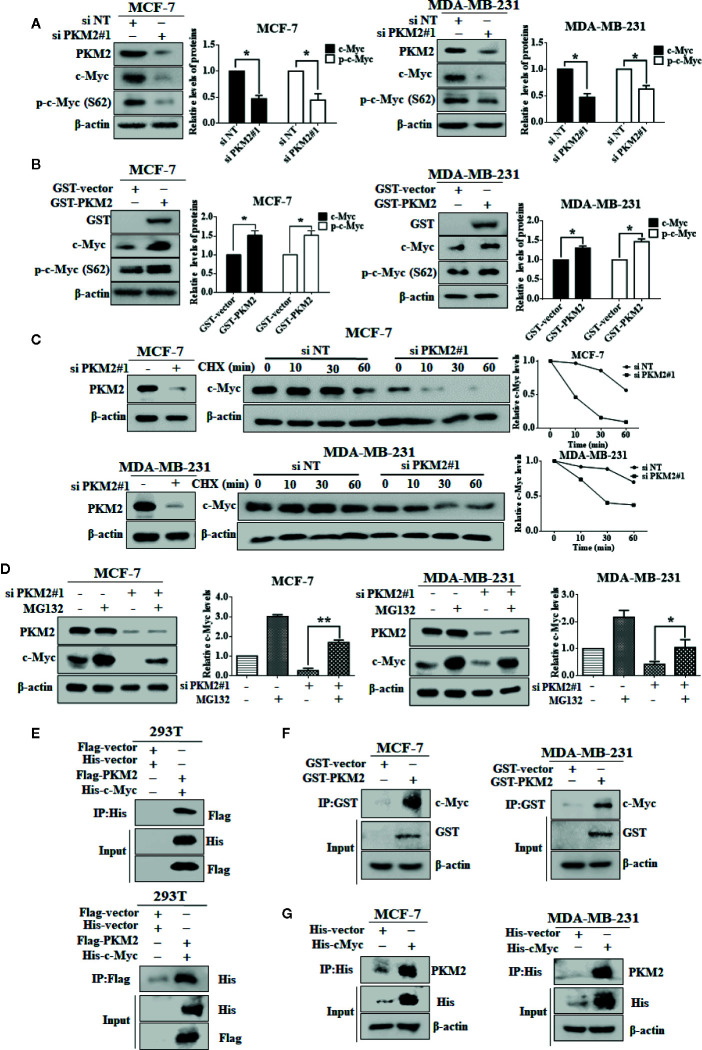
PKM2 enhances c-Myc stability by inducing the phosphorylation of c-Myc. **(A)** MCF-7 or MDA-MB-231 cells were transfected with either the non-targeting siRNA or PKM2 siRNA. PKM2, c-Myc and p-c-Myc (Ser62) protein levels were measured by immunoblotting. *β*-actin served as a loading control. **(B)** MCF-7 or MDA-MB-231 cells were transfected with either the empty vector plasmid or GST-PKM2 plasmid. PKM2, c-Myc and p-c-Myc (Ser62) protein levels were measured by immunoblotting. *β*-actin served as a loading control. Data were shown as the mean ± SD of three independent experiments (*p < 0.05, **p < 0.01). **(C)** sMCF-7 or MDA-MB-231 cells were transfected with either the non-targeting siRNA or PKM2 siRNA, and were treated with CHX (10 μg/ml) for the indicated time, and immunoblotting analysis was applied to detect the expression levels of c-Myc. *β*-actin served as a loading control. **(D)** MCF-7 and MDA-MB-231 cells were transfected with either the non-targeting siRNA or PKM2 siRNA, and were treated with MG132 (20 μM) before extracting proteins. Western blotting was used to analyze PKM2 and c-Myc proteins in MCF-7 and MDA-MB-231 cells. *β*-Actin was used as a loading control. **(E)** 293T cells were transfected with Flag-PKM2 plasmid and His-c-Myc plasmid, and then subjected to immunoprecipitation with anti-His and anti-flag antibody. The lysates and immunoprecipitation were then analyzed. **(F)** MCF-7 or MDA-MB-231 cells were transfected with either the empty vector plasmid or GST-PKM2 plasmid, and then subjected to immunoprecipitation with an anti-GST antibody. The lysates and immunoprecipitation were analyzed. **(G)** MCF-7 or MDA-MB-231 cells were transfected with either the empty vector plasmid or His-c-Myc plasmid, and then subjected to immunoprecipitation with an anti-His antibody. The lysates and immunoprecipitation were analyzed.

We next examined the relationship between PKM2 and c-Myc. After transfected with PKM2 siRNA, a significant decrease of c-Myc protein expression can be observed in MCF-7 and MDA-MB-231 cells (**Figure 5A**), and the increase of c-Myc protein level can be observed after transfected with exogenous PKM2 (**Figure 5B**). Moreover, we found that the expression of p-c-Myc (Ser-62) was decreased or increased when transfected with PKM2 siRNA or GST-PKM2 plasmid in MCF-7 and MDA-MB-231 cells. It has been previously demonstrated that the phosphorylation of c-Myc on Ser-62 results in its stabilization (Seo et al., 2008). Thus, we wanted to know whether the regulation of PKM2 on c-Myc phosphorylation will stabilize c-Myc protein. We next examined the degradation rate of the c-Myc protein by CHX assay in MCF-7 and MDA-MB-231 cells. As shown in **Figure 5C**, PKM2 knockdown significantly shortened the half-life of c-Myc both in MCF-7 and MDA-MB-231 cells. In line with the results in **Figure 5C**, we found that MG132, a proteasome inhibitor, could rescue the down-regulation of c-Myc in the cells with knockdown of PKM2 expression (**Figure 5D**). Next, we further examined the physical interaction between PKM2 and c-Myc proteins. 293T cells transfected with Flag-PKM2 plasmid and His-c-Myc plasmid were subjected to immunoprecipitation with an anti-His antibody, and Flag-PKM2 was detected. Similarly, His-c-Myc could be detected in immunoprecipitation complexes when the anti-flag antibody was used for immunoprecipitation (**Figure 5E**). Additionally, MCF-7 and MDA-MB-231 cells were transfected with GST-vector or GST-PKM2, then were subjected to immunoprecipitation with an anti-GST antibody, and c-Myc was presented in anti-GST co-IPs from cells transfected with GST-PKM2, but not in the cells transfected with GST-vector (**Figure 5F**). Similarly, MCF-7 and MDA-MB-231 cells were transfected with His-vector or His-c-Myc, then were subjected to immunoprecipitation with an anti-His antibody, and PKM2 was presented in anti-His co-IPs from cells transfected with His-c-Myc (**Figure 5G**), suggesting the interaction between PKM2 and c-Myc. These results for the first time brought our attention that phosphorylation of c-Myc at Ser-62 by PKM2 promoted the stability of c-Myc.

### Suppression of PKM2 Enhanced Tamoxifen Sensitivity in MCF-7 and MCF-7/TAMR Cells

**Figure 6:**
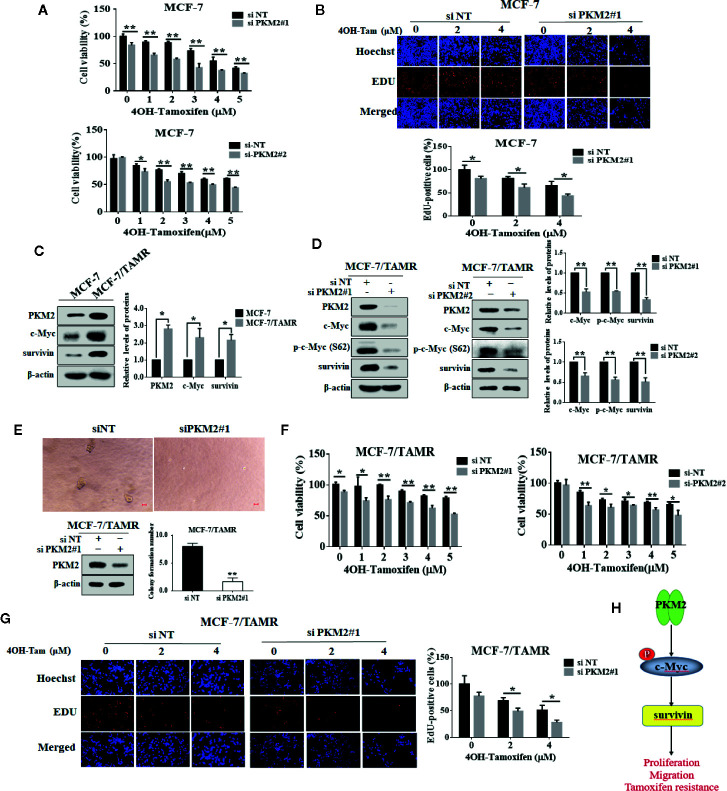
PKM2 downregulation enhanced cell sensitivity to tamoxifen in MCF-7 and MCF-7/TAMR cells. The MCF-7 cells were transfected with siPKM2 or non-targeting siRNA. Then the cells were treated with 4-OHT for 72 h at the indicated concentration periods (0–5 μM). **(A)** Cell viability was measured by CCK-8 reagent. **(B)** Cell proliferation ability was measured by EdU. Magnification, ×200. **(C)** Western blotting was used to analyze PKM2, c-Myc and survivin proteins in tamoxifen resistant and their parental cells. *β*-Actin was used as a loading control. **(D)** PKM2 siRNA-treated and control siRNA-treated MCF-7/TAMR cells were treated for 48 h. Western blot was performed with indicated antibodies. **(E)** The anchor-independent cell growth ability of MCF-7/TAMR cells transfected with PKM2 siRNA was detected by soft agar clone formation assay. MCF-7/TAMR cells were cultured for 10 days. The clone size greater than 50 μm is considered a clone formation. The MCF-7/TAMR cells **(F, G)** were transfected with siPKM2 or non-targeting siRNA. Then the cells were treated with 4-OHT for 72 h at the indicated concentration periods (0–5 μM). **(F)** Cell viability was measured by CCK-8 reagent. **(G)** Cell proliferation ability was measured by EdU. Magnification, ×200. **(H)** A schematic model of PKM2–c-Myc–survivin axis leading to proliferation, migration and tamoxifen resistance. The results are reported as the mean ± SD of triplicate measurements; *P < 0.05, **P < 0.01, t-test, siNT vs. siPKM2.

We further investigated whether PKM2 could be related to the regulation of tamoxifen sensitivity in MCF-7 cells. As shown in **Figures 6A, B**, PKM2 knockdown decreased the cell viability and proliferation in MCF-7 cells with 4OH-Tamoxifen treatment. Furthermore, we found that the expressions of PKM2, c-Myc and survivin were upregulated in MCF-7/TAMR cells as compared to MCF-7 cells (**Figure 6C**). Silencing of PKM2 resulted in a prominent decrease in the levels of c-Myc, p-c-Myc (Ser62) and survivin in MCF-7/TAMR cells (**Figure 6D**). To further investigate the role of PKM2 in MCF-7/TAMR cell proliferation, the effect of PKM2 on the anchorage-independent cell growth was measured by soft agar colony formation assay. As shown in **Figure 6E**, the size and number of clones were significantly decreased after PKM2 was silenced. We next asked whether silencing the expression of PKM2 could re-sensitize tamoxifen resistant breast cancer cells to tamoxifen treatment. **Figures 6F, G** showed that the cells became more sensitive to tamoxifen after knocking down PKM2. This result indicates that PKM2-c-Myc-survivin pathway participated in the regulation of tamoxifen resistance (**Figure 6H**), and knockdown of PKM2 enhanced cell sensitivity to tamoxifen in MCF-7 and MCF-7/TAMR cells.

### Elevated Levels of PKM2, Survivin, and c-Myc Correlate With Poor Relapse-Free Survival in Patients With ER+ Breast Cancer Undergoing Tamoxifen Therapy

**Figure 7:**
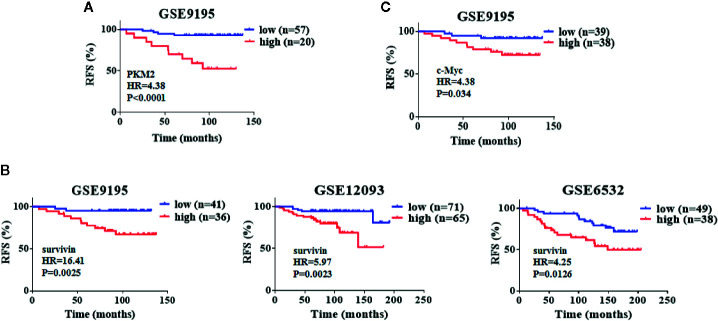
Elevated levels of PKM2, survivin and c-Myc correlate with poor relapse-free survival in patients with ER+ breast cancer undergoing tamoxifen therapy. **(A)** Kaplan–Meier analysis with auto select best cutoff values of PKM2 expression for ER+ breast cancer patients treated with tamoxifen from GEO datasets. **(B)** Kaplan–Meier analysis with auto select best cutoff values of survivin expression for ER+ breast cancer patients treated with tamoxifen from GEO datasets. **(C)** Kaplan–Meier analysis with auto select best cutoff values of c-Myc expression for ER+ breast cancer patients treated with tamoxifen from GEO datasets. P-values were calculated by the log–rank test.

To examine the clinical relevance of our result, we investigated three publicly available microarray datasets, which include the relapse free survival of patients in ER+ positive breast cancer patients treated with tamoxifen. In these datasets, we analyzed the survival rates applying the Kaplan–Meier method by a log-rank test, which indicate that PKM2, survivin and c-Myc overexpression confer a high risk of relapse in breast cancer patients treated with tamoxifen (**Figures 7A–C**). These data suggest that the PKM2, survivin and c-Myc expression levels may be important prognostic factors for tamoxifen treatment in breast cancer patients.

## Discussion

Breast cancer is the most common malignancy and the second leading cause of cancer-related mortality among women (Urun et al., 2015). Tamoxifen, a selective ER modulator, competitively restraints the binding of estradiol to ER, as a result inhibiting the ER-mediated transcription of kinds target genes to repress the proliferation of cancer cells. Although it is effective in adjuvant and first-line treatment of advanced ESR-positive breast cancer, development of resistance to tamoxifen remains a serious clinical problem (Nass and Kalinski, 2015). Therefore, it is imperative to find novel targets in breast cancer progression and improve breast cancer response to tamoxifen therapy. In this study, we reported for the first time that PKM2-c-Myc-survivin signaling cascade promoted breast cancer cell proliferation, migration, tamoxifen resistance (**Figure 6H**), and inhibition of PKM2 not only blocked cancer progression, but also enhanced tamoxifen efficacy in MCF-7 and MCF-7 resistant cells.

Accumulating evidence has demonstrated the important role of PKM2 in promoting cancer progression (Zheng et al., 2018; Liu et al., 2019). Consistently, we presented evidence that PKM2 promoted breast cancer cell proliferation and migration, and PKM2 overexpression predicted poor prognosis in breast cancer patients. These results revealed that PKM2 is a potential target for the treatment of breast cancer. It has been previously shown that glycolytic enzyme PKM2 which PKM2/NF-*κ*B/miR-148a/152 feedback circuit can regulate breast cancer cells growth and angiogenesis (Yan et al., 2017), but the regulatory mechanism of PKM2 as a protein kinase on breast cancer cell proliferation and migration remains to be further explored. It was reported that the PKM2–β-catenin interaction leaded to increased binding of *β*-catenin to the promoter region of c-Myc (Yang et al., 2011). Our study provided new insight in the mechanistic regulation of PKM2 in c-Myc, that PKM2 interacted with c-Myc and regulated c-Myc phosphorylation, providing the first evidence that c-Myc may be a novel substrate of PKM2. We found that inhibition of PKM2 decreased c-Myc phosphorylation, resulting in down-regulating c-Myc protein expression by promoting its degradation. As an oncoprotein, c-Myc promotes cancer progression by increasing the transcription of substrate genes involved in the control of cell proliferation or growth. It has been reported that c-Myc can promote PKM2 mRNA expression by upregulation of heterogeneous nuclear ribonucleoprotein (hnRNP) transcription (David et al., 2010). Consistent with the report, we found that overexpression of c-Myc increased the expression of PKM2 compared with PKM2 knockdown cells, indicating that there is a positive feedback loop between PKM2 and c-Myc. Database analyzation and experimental results revealed the positive regulation of PKM2 on survivin transcription. As a transcription factor, c-Myc regulates survivin transcription. We verified that overexpression of c-Myc abrogated the decrease of mRNA and protein levels of survivin induced by PKM2 inhibition. Taken together, we concluded that PKM2 regulated survivin through c-Myc. Our results revealed that PKM2–c-Myc–survivin cascade promotes the proliferation and migration of breast cancer cells, serving as a potential therapeutic strategy in breast cancer.

Accumulating evidence indicates that PKM2 is highly correlated with drug resistance (Li et al., 2015; Wang et al., 2018). However, no clear evidence reveals the role of PKM2 in the development of tamoxifen resistance. Our findings showed that PKM2 was upregulated in the tamoxifen resistant breast cancer cells compared to sensitive cells. Inhibition of PKM2 significantly decreased c-Myc and survivin expressions. As previously reported, c-Myc and survivin were related with the development of tamoxifen resistance. It was reported that aspirin can down-regulate c-Myc protein expression to overcome tamoxifen resistance (Cheng et al., 2017). Wen-Tsung Huang demonstrated that survivin was overexpressed in MCF-7/TAMR cells as compared to MCF-7 cells, and down-regulation of survivin restored the sensitivity of MCF-7/TAMR cells to tamoxifen. Our data showed that down-regulation of PKM2 not only rendered MCF-7 cells more sensitivity to tamoxifen, but also significantly overcame tamoxifen resistance in MCF-7/TAMR cells. The implication of PKM2 in enhancing tamoxifen sensitivity was further verified in breast cancer patients that the high expression of PKM2 confers a high risk of recurrence or relapse in patients treated with tamoxifen. Collectively, PKM2 may serve as a unique therapeutic target for overcoming tamoxifen resistance in breast cancers. This also provides a hint that PKM2 inhibitors combined with endocrine drugs may be a new strategy for the treatment of tamoxifen resistance in breast cancer patients.

Taken together, the present study demonstrated for the first time that PKM2-dependent c-Myc-Ser-62 phosphorylation stabilized c-Myc, thereby increasing survivin expression, which is required for breast cancer cell proliferation and migration. Inhibition of PKM2 blocked breast cancer progression and sensitized breast cancer cells to tamoxifen, indicating that PKM2 inhibitor may be an effective combination treatment in breast cancer patients treated with tamoxifen. Altogether, PKM2 represents a strong predictor for poor prognosis and drug resistance in breast cancer and targeting PKM2–c-Myc–survivin cascade could be a novel therapeutic strategy for breast cancer treatment, even tamoxifen resistant breast cancer.

## Data Availability Statement

The raw data supporting the conclusions of this article will be made available by the authors, without undue reservation.

## Author Contributions

PY and A-XL designed the study. PY, A-XL, X-SC, MT, and H-YW performed the experiments. PY, A-XL, X-LW, YZ, and K-SW collected, analyzed, and interpreted the data. A-XL and YC prepared the manuscript. All authors contributed to the article and approved the submitted version.

## Conflict of Interest

The authors declare that the research was conducted in the absence of any commercial or financial relationships that could be construed as a potential conflict of interest.

## Supplementary Material

Supplemental Figure 1
**(A)** GEPIA analysis showed the expression levels of PKM2 between ER+ and triple negative breast cancer tissues. **(B)** Western Blot was used to analyze PKM2 proteins in MCF-7, MDA-MB-231, and BT549 cells. *β*-Actin was used as a loading control.
