# Supplementary figures and images for: PKM2–c-Myc–Survivin Cascade Regulates the Cell Proliferation, Migration, and Tamoxifen Resistance in Breast Cancer
Source: Front Pharmacol. 2020 Sep 8;11:550469. doi: 10.3389/fphar.2020.550469 (PMC7506054; doi:10.3389/fphar.2020.550469)

## Slide 1
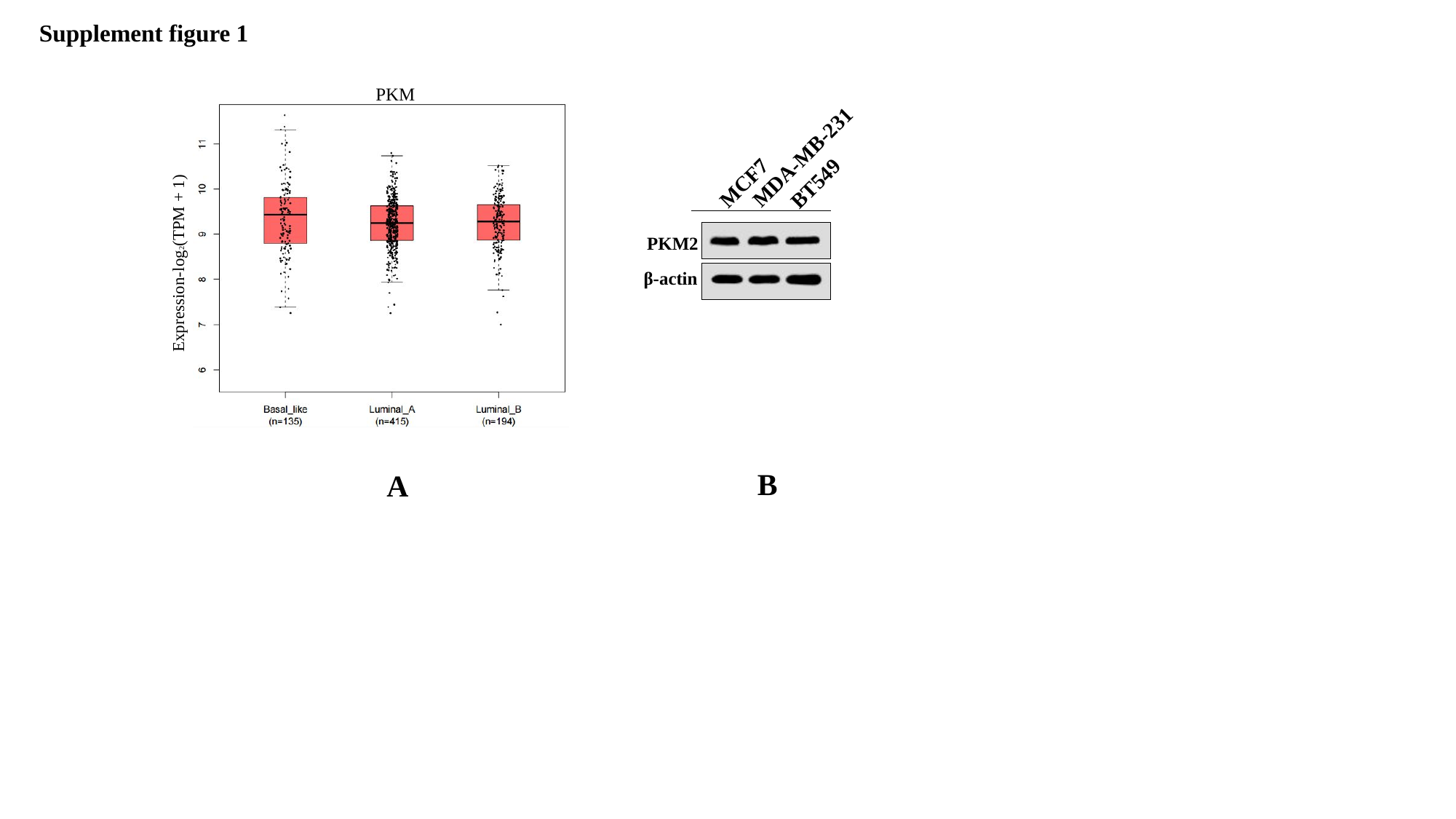

Supplement figure 1
PKM
MDA-MB-231
MCF7
BT549
PKM2
β-actin
Expression-log2(TPM + 1)
B
A

Supplement: Supplemental Figure 1 — (A) GEPIA analysis showed the expression levels of PKM2 between ER+ and triple negative breast cancer tissues. (B) Western Blot was used to analyze PKM2 proteins in MCF-7, MDA-MB-231, and BT549 cells. β-Actin was used as a loading control. [file Presentation_1.pptx]
